# Investigating the Long-term Durability of Prosthetic Valves in Patients Who Have Undergone Tetralogy of Fallot Surgery with Transannular Patch

**DOI:** 10.1093/icvts/ivaf159

**Published:** 2025-07-04

**Authors:** Gianluca Brancaccio, Marin Verrengia, Matteo Trezzi, Veronica Bordonaro, Ileana Croci, Enrico Piccinelli, Fabio Miraldi, Marcello Chinali, Aurelio Secinaro, Victoria D’Inzeo, Roberta Iacobelli, Lorenzo Galletti

**Affiliations:** Pediatric Cardiac Surgery Unit, Bambino Gesù Children’s Hospital, IRCCS, Piazza S. Onofrio, 4, 00165, Rome, Italy; Department of Cardiac Surgery, University “La Sapienza” of Rome, Via del Policlinico 155, 00187, Rome, Italy; Pediatric Cardiac Surgery Unit, Bambino Gesù Children’s Hospital, IRCCS, Piazza S. Onofrio, 4, 00165, Rome, Italy; Advanced Cardiothoracic Imaging Unit, Bambino Gesù Children’s Hospital, IRCCS, Piazza S. Onofrio, 4, 00165, Rome, Italy; Predictive and Preventive Medicine Research Unit, Bambino Gesù Children’s Hospital, IRCCS, Piazza S. Onofrio, 4, 00165, Rome, Italy; Pediatric Cardiology Unit, Bambino Gesù Children’s Hospital, IRCCS, Piazza S. Onofrio, 4, 00165, Rome, Italy; Department of Cardiac Surgery, University “La Sapienza” of Rome, Via del Policlinico 155, 00187, Rome, Italy; Pediatric Cardiology Unit, Bambino Gesù Children’s Hospital, IRCCS, Piazza S. Onofrio, 4, 00165, Rome, Italy; Advanced Cardiothoracic Imaging Unit, Bambino Gesù Children’s Hospital, IRCCS, Piazza S. Onofrio, 4, 00165, Rome, Italy; Pediatric Cardiac Surgery Unit, Bambino Gesù Children’s Hospital, IRCCS, Piazza S. Onofrio, 4, 00165, Rome, Italy; Pediatric Cardiology Unit, Bambino Gesù Children’s Hospital, IRCCS, Piazza S. Onofrio, 4, 00165, Rome, Italy; Pediatric Cardiac Surgery Unit, Bambino Gesù Children’s Hospital, IRCCS, Piazza S. Onofrio, 4, 00165, Rome, Italy

**Keywords:** pulmonary valve replacement, valve prosthesis, homograft, tetralogy of Fallot

## Abstract

**Objectives:**

This study aimed to determine the longevity of bioprosthetic valves (BPVs) or valved conduits in the pulmonary position and the factors associated with prosthetic valve longevity in patients who underwent surgical repair of tetralogy of Fallot (TOF).

**Methods:**

We performed a single-centre retrospective study in patients undergoing placement of a bioprosthesis or valved conduit in patients operated for TOF with a transannular patch. The primary end-point was freedom from pulmonary valve replacement (PVR) reintervention. The composite secondary end-point was freedom from PVR reintervention and structural valve degeneration.

**Results:**

A total of 211 patients undergoing 255 operations were identified. The median age at first PVR was 15.4 years (IQR 12.3-20.7 years). The freedom from reintervention at 5, 10, and 15 years was 94%, 77%, and 64%, respectively. Multivariable analysis shows that factors that are independently associated with a more significant hazard of reintervention are younger age, smaller valve size, and male sex. Of the implanted prosthetic valves, 122 were bioprosthesis; 38 (18%) were homografts; 37 (17%) percutaneous valve, and 14 (7%) were other prosthetic valves or conduits. The freedom from reintervention was not different between homografts and all other heterografts (*P* = 0.938). Percutaneous valves showed an average longevity of 9.4 years, significantly lower than other prosthetic valves (*P* = 0.007).

**Conclusions:**

Younger patient age and a smaller valve size were associated with reduced BPV durability. There is a concern about an early PVR to preserve right ventricle function overall during adolescent age.

## Graphical abstract

**Figure:**
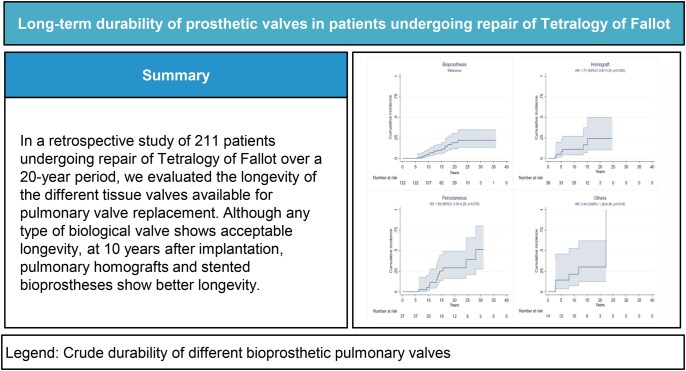

## INTRODUCTION

Tetralogy of Fallot (TOF) is a cyanotic congenital heart disease (CHD) with a multifactorial aetiology, accounting for 7%-10% of all congenital cardiac malformations.^1^

The surgical repair includes patients with a small pulmonary annulus correction with a transannular patch to relieve the right ventricular (RV) outflow tract obstruction, and the common sequela of this procedure is pulmonary valve (PV) regurgitation. Pulmonary regurgitation is initially well tolerated; however, over the years, RV volume overload has led to RV dilatation and subsequent symptoms of RV failure, life‐threatening arrhythmias, and reduced survival.^2^^,^^3^ Although PV replacement (PVR) is the standard treatment for this condition, controversy exists about which prosthesis should be used. Homografts have been considered the first choice for a long time.^4–6^ Despite stented bioprosthesis being readily available, being more accessible to be implanted, and having shown excellent results in the aortic position, their durability for PVR remains controversial.^7^ Mechanical prostheses are rarely used because of the increased risk of thrombosis in PV position and patient’s choice.^3^ Long‐term durability of which prosthesis should be preferred for PVR in patients with TOF is analysed, comparing long‐term results after PVR with different prostheses using a 20‐year single‐centre cohort.

## METHODS

### Ethical statement

The study conforms to the ethical principles of WMA Declaration of Taipei and is in compliance with the current Italian regulations. The IRB was notified on December 3, 2024. Individual consent waived due to retrospective design and anonymity (HCRW) (IRAS ID: 257758, July 23, 2019).

### Study population and follow-up

We examined the records of all patients undergoing placement of a bioprosthesis or valved conduit in the pulmonary circulation after TOF repair with a transannular patch between June 2007 and December 2023 using the Bambino Gesù Children’s Hospital database.

A total of 211 patients underwent 255 PVRs. The type of implanted valve was chosen based on the surgeon’s assessment and homograft availability. Patients with double outlet right ventricle or pulmonary atresia with ventricular septal defect (VSD) and TOF with absent PV were excluded from the analysis, such as patients corrected with valved conduit and not with a transannular patch to avoid any potential effects on PV durability and the choice of valve type.

Demographics collected include date of birth, sex, genetic syndrome, intraoperative weight, and height at the time of PVR and body surface area (BSA). The last date of follow-up record included in this study was April 1, 2024. Follow-up includes the date of the most recent follow-up, echocardiogram reports, and cardiac magnetic resonance imaging (CMRI) reports obtained as part of a routine examination. More specifically, data from echocardiogram reports will include estimated RV pressure gradient, maximum right ventricular outflow (RVOT) pressure gradient, and degree of pulmonary stenosis and regurgitation to evaluate structural valve degeneration (SVD) and degree of tricuspid valve (TV) regurgitation and RV dysfunction. For CMRIs, we analysed the most recent preoperative CMRI and the most recent postoperative CMRI, both conducted within 6 months of the corresponding procedure, as well as the final CMRI available at follow-up. For echocardiograms, we evaluated the last echocardiogram performed immediately prior to reintervention and the final echocardiogram available at follow-up.

Postoperative antithrombotic management was tailored to the type of valve and patient-specific risk factors. Patients receiving bioprosthetic valves (BPVs) were routinely prescribed low-dose aspirin for a minimum of 6 months.

### Indications for PVR

The indications of operation were symptoms and RV enlargement. For asymptomatic patients, we have maintained a proactive approach towards PVR as evidenced by the median age of 15 years at PVR. The median RV end-diastolic and end-systolic volumes were 242.6 and 117.5 mL/m^2^, respectively. Since 2013, based on the findings of previous study,^3^^,^^11^ we used the RV end-diastolic volume of 160 mL/m^2^ and RV end-systolic volume of 80 mL/m^2^ as a guide to assist decisions regarding PVR in the asymptomatic patients.

### End-point

The primary end-point was to compare long‐term freedom from PVR reintervention. The secondary end-point was to assess long-term freedom from both PVR reintervention and SVD, reflecting the durability and sustained performance of the valve prosthesis. At echocardiographic examination, SVD was defined as a transpulmonary valve peak systolic pressure decrease of ≥50 mmHg or significant pulmonary regurgitation (3+).^7^^,^^9^^,^^10^ Information about reintervention and SVD was obtained from outpatient and inpatient clinical letters. Moreover, existing echocardiographic examination reports were used to compare echocardiographic measurements in all patients at the latest follow‐up echocardiographic examination. Velocity across the PV was derived from continuous wave Doppler echocardiography, and peak systolic pressure gradient was estimated using the modified Bernoulli equation. Pulmonary regurgitation was graded into 5 categories: 0, absent; 1, trivial; 2, mild; 3, moderate; and 4, severe. Finally, to determine the effect of PVR on RV volume (indexed for BSA) changes after surgery, data from available preoperative and follow‐up cardiac MRI examinations were reviewed and analysed.

### Statistical analysis

Continuous variables were reported as median and interquartile range (IQR) because they did not follow a normal distribution. Normality was assessed using the Shapiro-Wilks test. Comparisons of patients’ characteristics by valve type were performed using the Kruskal-Wallis test, while comparisons of CMR characteristics before and after surgery were conducted using the Wilcoxon signed-rank test for paired data. Categorical variables were tabulated as frequencies and percentages and compared using the Fisher’s exact test. A conditional Cox proportional hazards model was used to account for specific subgroups and minimize potential biases related to patient selection. Variables included in the model were selected based on their clinical relevance, previous literature, and univariate analysis (*P* < 0.10) to identify potential risk factors for reintervention. The primary end-point was defined as freedom from PVR reintervention, while the secondary end-point included freedom from both PVR reintervention and SVD. Receiver operating characteristic (ROC) analysis was used to assess the discriminative ability of age at surgery as a predictor of reintervention. The area under the curve (AUC) was calculated, and the 95% confidence interval (CI) was reported to evaluate the predictive power. Similarly, Kaplan-Meier survival curves and competing risk analysis were generated to estimate the incidence of redo PVR and to estimate valve durability over time. Statistical analyses were performed using SPSS version 21.0 statistical software (IBM, USA).

## RESULTS

### Study population

The median age of the entire cohort at first PVR was 15.4 years (IQR: 12.3-20.7 years). The general characteristics of the population are summarized in **Table 1**. Approximately 18% and 2% of the patients underwent at least 2 and 3 PVR procedures, respectively. The devices implanted were grouped into 4 major categories (stented bioprosthesis, homograft, percutaneous valve, and other valved conduits), as shown in **Table 2**. The category “other valved conduits” includes Contegra [Medtronic] valved conduit or Hancock [Medtronic] conduit and other types of valved conduits currently no longer available. The choice of valve prosthesis varied across the age range of the cohort. In general, homografts were most commonly used in the youngest patients and approximately exclusively in patients under 6.

**Table 1.** Patient Characteristics

	Overall *n* = 211	Bioprosthesis *n* = 122	Homograft *n* = 38	Percutaneous *n* = 37	Others *n* = 14	*P*
Male (*n*, %)	141 (66.8)	83 (68.0)	24 (63.2)	24 (64.9)	10 (71.4)	0.923
Palliative shunt (*n*, %)	33 (15.7)	16 (13.2)	7 (18.4)	7 (18.9)	3 (21.4)	0.613
Age at shunt palliation, mo (median, IQR)	1.7 (0.4-5.0)	2.1 (0.2-5.0)	4.1 (0.8-7.2)	0.5 (0.4-1.7)	0.4 (0-5.4)	0.578
Age at transanular repair, mo (median, IQR)	9.1 (4.7-16.9)	9.8 (5.3-16.6)	7.0 (4.0-15.8)	7.1 (4.6-17.4)	12.7 (4.9-20.6)	0.542
Time from transanular repair to PVR, mo (median, IQR)	14.5 (11.6-19.0)	15.1 (12.1-19.4)	12.4 (5.9-16.2)	15.6 (12.1-20.3)	13.0 (8.3-18.0)	0.004
Patient age at implantation, y (median, IQR)	15.4 (12.3-20.7)	15.9 (13.1-22.1)	13.8 (7.4-17.6)	15.9 (12.4-21.7)	13.7 (8.6-22.7)	0.006
True inner valve diameter (median, IQR)	25 (24-27)	25 (25-27)	24 (22-26)	26 (23-26)	25 (22-29)	0.016

Abbreviations: IQR: interquartile range; PVR pulmonary valve replacement.

**Table 2.** Results of Cox Regression Analysis of Factors Influencing First PVR

Variables	HR (95% CI)	SE	*P*
Valve size at first PVR	0.78 (0.63-0.96)	0.106	0.021
Age at first PVR	0.93 (0.86-0.99)	0.036	0.044
Female (ref: Male)	0.31 (0.11-0.83)	0.501	0.021
Previous palliation	0.52 (0.12-2.22)	0.746	0.381
Preoperative TR	0.75 (0.10-5.71)	1.035	0.790
Heterograft (Ref: Homograft)	0.22 (0.04-1.14)	0.827	0.074

Abbreviations: CI: confidence interval; HR: hazard ratio; PVR: pulmonary valve replacement; TR: tricuspid regurgitation.

### Reintervention rates

Of the 211 cases, 39 underwent a surgical (*n* = 17) or cardiac catheterization (*n* = 22) reintervention to replace the PV in the native RVOT. The risk of reintervention was 2.38 events per 100 patient-years. The freedom from reintervention at 5, 10, and 15 years was 94%, 77%, and 64%, respectively. The freedom from SVD at 5, 10, and 15 years was 90%, 62%, and 40%, respectively.

### Reintervention rates by age

Time to reintervention differed significantly by age at surgery (*P* < 0.001). The risk of reintervention was approximately 5 times greater for children than adults (HR = 5.37; 95% CI, 2.23-12.89). Regardless of age, almost all events occurred more than 5 years after the primary valve replacement. After 5 years, the differences by age emerged. The 10-year reintervention rate was 41% for patients under 12 years, 22% for patients aged 12-19, and 7% for patients aged 19 or older. The ROC curve for reintervention demonstrated that younger age had a high predictive power (AUC: 0.715, SE: 0.047, 95% CI, 0.649-0.774) with age less than 14 years (Youden index: 0.347).

### Reintervention rates by valve

**Figure 1.**
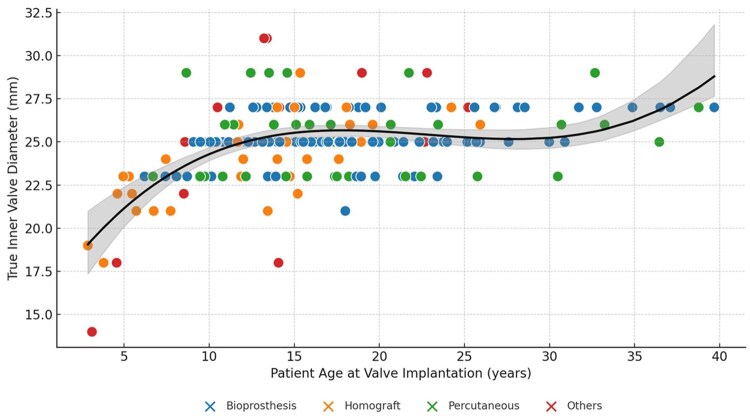
Distribution of Different Prosthesis at the PVR

**Figure 2.**
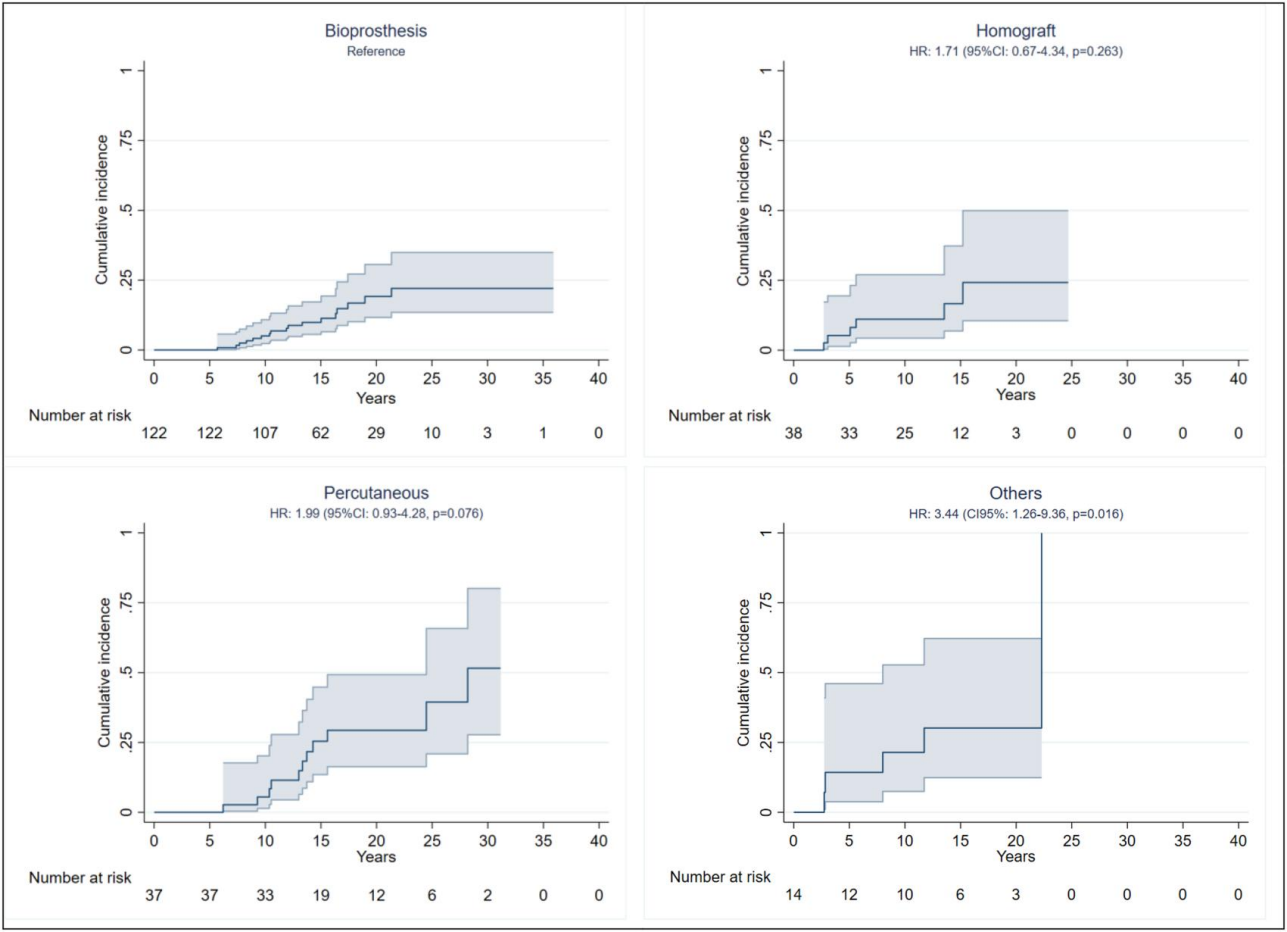
Crude Durability of Different Bioprosthetic Pulmonary Valves. Abbreviations: CI: confidence interval; HR: hazard ratio

Operative PVR data are summarized in **Table 2**. Three types of stented BPVs were surgically implanted: the Carpentier Edwards (CE; Model 2700; Edwards Lifesciences, Irvine, Calif) Magna Ease valve (Model 3000 TFX); the Medtronic (Medtronic, Milan, Italy) Hancock II valve; and the CE Inspiris Resilia (Model 2625) valve (**Figure 1**). PVR was performed directly into the native RVOT with an anterior bovine pericardium patch. Homografts used in our department are cryopreserved and implanted using the total pulmonary root replacement technique with concomitant reconstruction of pulmonary arterial confluence. Three percutaneous valves were inserted during cardiac catheterization: Melody [Medtronic] stented bovine jugular vein valve, SAPIEN [Edwards]valve, and the Venus P valve. All patients survived to discharge. The causes for prosthesis reintervention were stenosis (*n* = 19 pts), regurgitation (*n* = 9 pts), steno-insufficiency (*n* = 8 pts), and endocarditis (*n* = 3 pts). The overall crude median BPV durability was 18.6 years (15, 9-23 years). Of the implanted BPV, 122 were stented bioprosthesis (58%; CE Magna Ease = 80; CE Resilia = 22; Hancock II = 17); 38 (18%) were homografts; 37 (17%) percutaneous valve (Melody [Medtronic] *n* = 1, SAPIEN [Edwards]: *n* = 34); and 14 (7%) were other BPV. The cumulative incidence of replacement 10 years after implantation was 5% for bioprosthesis, 11.1% for homografts, 5.5% for percutaneous valves, and 21.4% for other heterografts (**Figure 2**). In our study, the type of valve (*P* = 0.007) influences the longevity and durability of the valves; in particular, the percutaneous valve shows a risk 3 times greater to be reoperated to respect bioprosthesis (HR = 3.27; 95% CI, 1.19-8.98) and homografts (HR = 3.19; 95% CI, 1.03-9.85). The freedom from reintervention was not different between homografts and all other heterografts (*P* = 0.938). In the subanalysis of patients who underwent bioprosthesis implantation (*n* = 122), we observed no significant difference in terms of risk of reoperation. However, considering a composite end-point redo/SVD, there is a significant difference (*P* < 0.001) between the type of prosthesis, with the Resilia valves with a consistent risk of early degeneration to respect pericardial and bovine valves (HR = 3.99, 95% CI, 2.01-7.92 and HR = 10.13, 95% CI, 2.77-36.99).

### Other univariate risk factors for reinterventions

**Figure 3.**
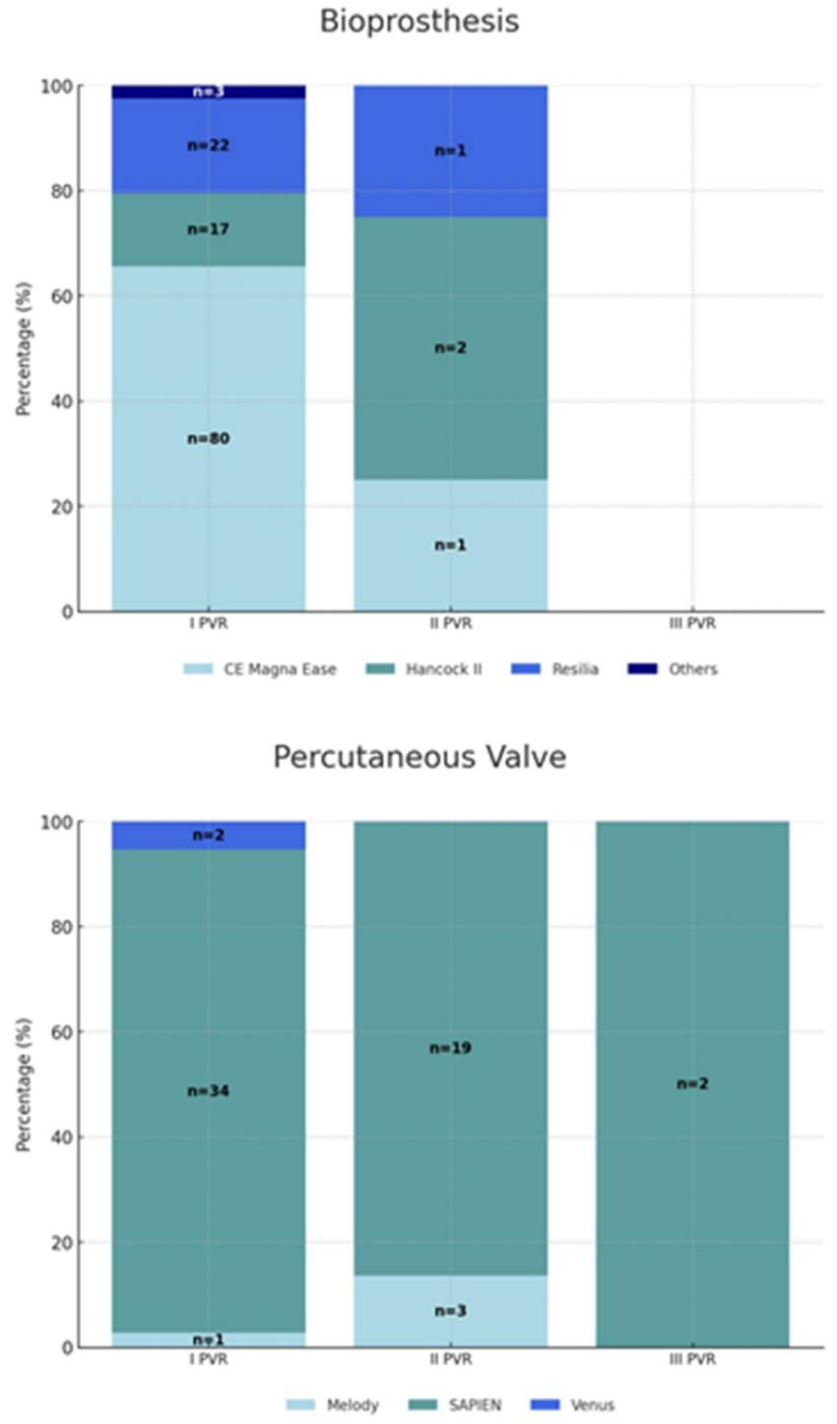
True Inner Diameter of the Implanted Prosthetic Pulmonary Valves According to the Patient Age at Implantation. Abbreviation: PVR: pulmonary valve replacement

Male sex and size smaller than 23 mm at first PVR are risk factors for reintervention (*P* = 0.040 and *P* = 0.033). The risk of reintervention was approximately 2-fold more significant in the male patients (HR = 1.98; 95% CI, 1.03-3.81), with a 10-year reintervention rate of 27% versus 18% in the female group. The prosthesis size plays a crucial role in the valve type decision-making; the valve size less than 23 mm shows a risk of reintervention 2 times greater concerning patients undergoing PVR with a larger prosthesis (HR= 2.10; 95% CI, 1.06-4.16). **Figure 3** illustrates the true inner diameter of the implanted BPV according to patient age at PVR, highlighting that the 25-mm diameter is the one most commonly used in PVR.

### Multivariable model for time to reintervention

A multivariable model was constructed to examine covariate-adjusted differences in time to reintervention in patients over 6 years to avoid any potential effect on the type of prosthesis choice. **Table 3** shows that factors that are independently associated with a more significant hazard of reintervention are younger age at first PVR, smaller valve size, and male sex. Previous palliation with Blalock-Taussig shunt tends to be protective against reintervention even if not significant (*P* = 0.790), but there is a strong correlation with RVEF at the time of first PVR (*P* < 0.001).

**Table 3.** CMR Characteristics in PVR in Pre and Post-op

Characteristics	Preoperative Median (IQR)	Postoperative Median (IQR)	*P*
RVEDV (mL)	239 (200-286)	203.3 (171.5-233.9)	<0.001
RDVEDVi (mL/m^2^)	160 (150-175)	116.3 (103-133.2)	<0.001
RVESV (mL)	115 (91-139)	99.3 (82.5-121.9)	<0.001
RVESVi (mL/m^2^)	78.2 (67.2-87.9)	57.7 (48.3-68)	<0.001
RF EF (%)	52 (48.3-56)	49.4 (46.3-53)	0.002
LVEDV (mL)	123 (101-152.3)	150.7 (125.3-181)	<0.001
LVEDVi (mL/m^2^)	84 (75-94)	89.6 (77.8-98.8)	0.008
LVESV (mL)	54 (41.2-69)	68.7 (52-84)	<0.001
LVESVi (mL/m^2^)	36 (29.9-42.1)	38.6 (31.6-46.3)	0.067
LV EF (%)	57 (53-61.9)	56 (53-59.8)	0.834
PR (%)	48 (39-54)	9.8 (3.9-18.7)	<0.001

Abbreviations: CMR: cardiac magnetic resonance; IQR: interquartile range; PVR: pulmonary valve replacement; RVEDV: right ventricular end diastolic volume; RDVEDVi: right ventricular end diastolic volume indexed; RVESV: right ventricular end systolic volume; RVESVi: right ventricular end systolic volume indexed; RF EF: right ventricular ejection fraction; LVEDV: left ventricular end diastolic volume; LVEDVi: left ventricular end diastolic volume indexed; LVESV: left ventricular end systolic volume; LVESVi: left ventricular end systolic volume indexed; LV EF: left ventricular ejection fraction; PR: pulmonary regurgitation.

## DISCUSSION

PVR has become one of the most commonly performed procedures in congenital heart surgery. Despite the prevalence of this procedure, considerable uncertainty remains as to the indications and timing for surgery and which valve replacement option is most optimal.^8^^,^^9^ The main findings were as follows: (1) younger age at PVR, smaller valve size, and male sex were associated with reduced BPV durability; (2) the median BPV durability was 20 years; (3) there was no substantial significant variation in the durability of different BPV, except percutaneous valves; (4) we confirm concern regarding the Inspiris Resilia valve in the pulmonary position.^10–14^^,^^19^

### Risk factors for PVR

In line with previous studies, we found that younger age at PVR is associated with reduced BPV durability.^15^ Younger age is a vital risk factor for PVR, and we found that the risk of reintervention is 5 times greater for children than adults, decreasing by 10% for each increasing year of age at surgery. This result may be obvious, but we observe a significant variability in the time of PVR despite the homogeneous study group. The wide range of age at first PVR demonstrates 2 essential aspects: (1) different tolerability of patients to volume overload and (2) probable non-adherence to strict criteria of PVR. From this perspective, a close correlation between previous palliation and RVEF should be seen, where late correction in childhood may have increased the degree of RV hypertrophy and, thus, tolerability to PR. The criterion for PVR has been to follow the RVEDV, changing values with increasingly stringent criteria based on the literature.^16^^,^^17^ This has also resulted in anticipation of PVR, as evaluated in other reports. Indeed, the adolescent age is where the most significant number of procedures are observed. The cut-off of 14 years that we found in our and other’s studies highlights this concept well.^18^ This category must be focused on because the possible delay of PVR can mean a gain in prosthesis longevity of almost 6 years. Indeed, we suggest in this age group the possibility, even in the face of volumes above the cut-offs, of evaluating RVEF over time as a guide to the timing of PVR.

### BPV longevity

The longevity of almost 20 years of the different prostheses is higher than that observed in the different previously published reports, and most importantly, there is no difference by valve type, excluding the percutaneous valves.^20^ We did not find a difference in the longevity of homografts compared with other heterografts, even though the results are controversial.^21^ The average longevity of almost 22 years suggests that homografts were the best choice in children, where implantation is more feasible than in adults.^22^ The lower longevity (average in time of 9.4 years) reported by percutaneous valves reflects the outcome of TAVI versus bioprosthesis in the aortic position.^23^^,^^24^ At the same time, the advantage of these valves in avoiding a surgical approach is undeniable, reducing the risks of operative morbidity and mortality.^25^ The most significant concern always goes to the adolescent category, where the spread of percutaneous options opens scenarios of notable increases in procedures throughout life. If we postulate that the first PVR occurs on average at the age of 17 years, we will expect a patient to undergo approximately 4 additional interventions for PVR by the age of 70 years. The cumulative procedural risk and benefit must be carefully considered and extensively discussed with the patient’s family.

### Bioprosthesis valves

Variation in the performance of different BPVs is exciting because, unlike other factors, the BPV choice is modifiable. So far, only a few studies have reported significant differences in the durability of different BPVs.^26^^,^^27^ Our study’s average durability of almost 16 years explains well that it is our first choice for PVR in adolescent and adult populations. It is of great interest that pericardial and porcine valves show good outcomes. Conversely, we confirm the concerns about the [Edward] Inspiris Resilia valve in terms of longevity. Our study reports early degeneration of the prosthesis in an average time of 7 years after implantation. Previous reports have advocated different mechanisms to explain early degeneration in pulmonic position as follows: (1) wrong technical implantation; (2) early degeneration secondary to micro-thrombi apposition on leaflet during the postoperative period; (3) malfunctioning in the low-pressure system; (4) failure of anti-calcium treatment in the pulmonary circulation.^28^^,^^29^ The exact mechanism is unknown, but it is of interest the suggestion from the Mayo Clinic to use anticoagulation drugs after BPV implantation to delay the occurrence of SVD for almost 6 months after PVR.^30^

### Future perspectives

Developing serially expandable valves, particularly those made from materials like Gore-Tex or heart valves with regenerative properties based only on a biocompatible synthetic structure, represents a significant advancement in cardiac therapy, especially for paediatric patients.^31^ These “growing” valves are critical in the younger population due to their continued growth and the limitations of traditional valves that may require multiple replacement surgeries. Continued research and clinical trials will be crucial in validating and bringing these innovations to clinical practice.

### Study strengths and limitations

Important strengths of this study are the homogeneity cohort consisting exclusively of patients with TOF repaired with transannular patch and the extended follow-up. Because of the retrospective nature of this study, bias caused by unavailable data or unrecognized confounding may persist. This study was concerned with durability, which is measured as the time to replacement. However, BPV dysfunction may occur long before replacement is performed; thus, the provided durability estimates are likely inflated relative to durability measured as the time to BPV dysfunction. Despite thorough data collection efforts, a certain amount of data still needed to be included.

## CONCLUSIONS

PVR is one of the most performed procedures in congenital heart surgery. Younger patient age at PVR and a smaller valve size were associated with reduced BPV durability. There is a concern about an early PVR to preserve RV function overall in adolescent age, putting patients at risk for multiple procedures throughout life. Increasing percutaneous valve implantation at any age has reduced the postprocedural risk. However, the feasibility of performing multiple subsequent transcatheter valve-in-valve replacements in the same patient is still unknown and raises concerns for progressive patient-prosthesis mismatch.

## Data Availability

The derived data supporting this study’s findings are available from the corresponding author upon request.
